# Primary squamous cell carcinoma of the breast: a case report and review of literature

**DOI:** 10.11604/pamj.2015.20.152.6188

**Published:** 2015-02-18

**Authors:** Youssef Seddik, Sami Aziz Brahmi, Said Afqir

**Affiliations:** 1 Medical Oncology Department, University Hospital Mohammed VI, Oujda, Morocco

**Keywords:** Primary squamous cell carcinoma, breast cancer, aggressive tumor

## Abstract

Primary squamous cell carcinoma is a well known malignancy of the skin and other organs composed of squamous cells, which are normally not found inside the breast. Therefore, a primary squamous cell carcinoma of the breast is an exceedingly uncommon phenomen and the management of this type of disease is still unclear. We report the case of a 43-year-old Moroccan woman, without significant medical history, presented an infected mass of 9 cm in the left breast associated with ipsilateral axillary lymphadenopathy. The mass's surgical biopsy revealed a triple negative primary squamous cell carcinoma of the breast. She underwent a neoadjuvant chemotherapy using 5 Fluoro-Uracil and platinum. After three courses, she presented a contralateral breast progression and apparition of metastasis at D10. She received one course of a palliative chemotherapy based on weekly paclitaxel stopped because of her peformans status deterioration. She died 7 months after her admission.

## Introduction

Primary squamous cell carcinoma (PSCC) is a well known malignancy of the skin and other organs composed of squamous cells, which are normally not found inside the breast. Therefore, a primary squamous cell carcinoma of the breast is an exceedingly uncommon; it represents less than 0.1% of all breast carcinomas [1]. The diagnosis is established when squamous cell carcinoma is the only malignancy found in the breast specimen, metastases from another primary are excluded, and the tumor does not originate from the skin of the breast [2]. Because of the rarity of this neoplasm, data on its diagnosis, treatment and prognosis is limited to isolated case reports and case series. We report a case of PSCC of the breast and review of the literature.

## Patient and observation

**Figure 1:**
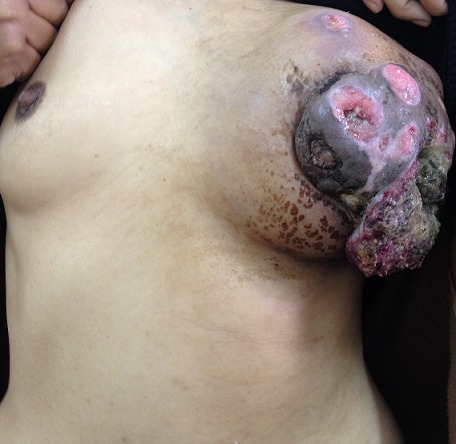
A left breast infected mass, measuring 9x8x9 cm in size, with some cystic areas

**Figure 2:**
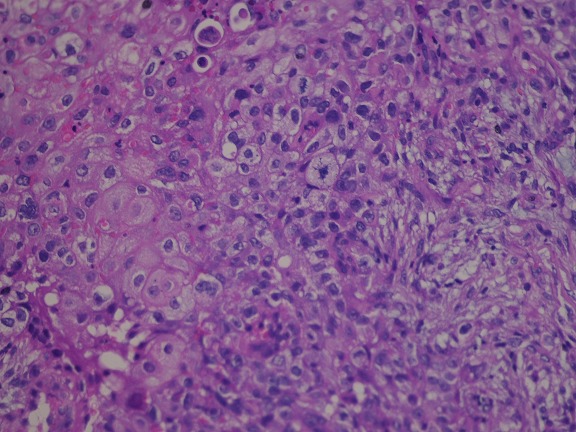
Squamous cell carcinoma of the breast

**Figure 3:**
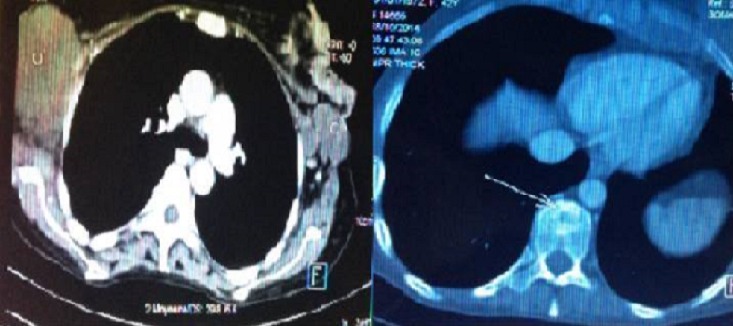
CT scan showed a contralateral breast progression and apparition bone metastatsis at D10

We report the case of a 43-year-old Moroccan woman, without history of skin or breast cancer, nor any skin, oro-pharynx, or anal lesions. Her family history was not significant for breast cancer. She was admitted to our center eight months ago for a mass in the left breast which had been growing rapidly. A physical examination revealed a left breast infected mass, measuring 9x8x9 cm in size, with some cystic areas, associated with ipsilateral axillary lymphadenopathy (Figure 1). Mammograhy demonstrated an irregular opacity with skin infiltration, approximately 9 cm in its greatest dimension, which was classified as BI-RADS 5. The right breast was negative on palpation and mammography. The mass's surgical biopsy revealed a primary squamous cell carcinoma of the breast (Figure 2). Immunohistochemical evaluation was negative for estrogen receptor (ER), progesterone receptor (PgR) and HER2. Metastatic disease was ruled out via Total body CT scan, and bone scintigraphy. The serum level of tumor marker CA 15-3 was 290 U/ml (≤ 30). She underwent three courses of neoadjuvant therapy: the first course using cisplatin (at a dose of 75 mg/m^2^ as a 1-hour intravenous infusion on day 1) and an infusion of 5-fluoro-Uracil as bolus (at a dose of 400 mg /m^2^ per day for 3 days) every 3 weeks. She presented a grade 3 vomiting and febril neutropenia which was treated by vancomycin with good evolution. Because of the side effects, we substitute, In the two others courses,cisplatin by Carboplatin (at an area under the curve of 5 mg per milliliter per minute, as a 1-hour intravenous infusion on day 1) and we substitute 5-fluorourcil bolus by continuous infusion (at a dose of 2400 mg/m^2^ for 2 days) every 3 weeks. The evaluation investigations based on CT scan showed a contralateral breast progression and apparition of bone metastatsis at D10 (Figure 3). She received a palliative chemotherapy based on weekly paclitaxel at a dose of 80 mg/m^2^, stopped after one course because of her general performans status deterioration. She died 7 months after her admission.

## Discussion

Primary squamous cell carcinoma (PSCC) of the breast is very rare. It represents less than 0.1% of all breast carcinomas [1]. Diagnosis is retained when no other neoplastic components such as ductal or mesenchymal elements are present in the lesion,the tumor origin is independent of the overlying skin and nipple and absence of an associated primary Squamous cell carcinoma in a second site (oral cavity bronchus, esophagus, renal pelvis, bladder, ovary, and cervix) [2]. The histogenesis of this malignancy is contreversial [3]. It has been suggested that it may be a very extreme form of squamous cell metaplasia, developing into an adenocarcinoma. Squamous cell metaplasia is also seen in cysts, chronic inflammations, ab¬scesses and adenofibromas. Therefore, PSCC is divided into pure squamous cell carcinoma and that mixed with adenocarcinoma. PSCC of the breast has been diagnosed in adult women of ages ranging from 29 years to 90 years [3]. No case was described in the male. The PSCC tend to be relatively large (> 4 cm) at diagnosis and cystic in 50% of the cases [1]. Some authors have noted only rare involvement of the lymph nodes [1–4]. There are no typical findings on the mammogram,ultrasound may show a complicated cyst or an inflammatory process [5]. Our patient was 43 year old, without significant medical history, she had an infected mass of 9cm associated with ipsilateral axillary lymphadenopathy, without typical findings on the mammogram. A surgical biopsy procedure is usually required to establish the diagnosis of squamous cell carcinoma which is generally characterized by histologically identified keratinization and intercellular bridges [6]. The breast Sqaumous cell carcinoma is generally a high-grade and triple negative tumor(ER, PR and HER2-negative) [1, 3, 7–9]. The immunohistology of our case is consistent with those findings. Ruohong Shui and collegues [10] reported three cases of primary squamous cell carcinoma of the breast with an unusual “basal-HER2” phenotype This tumor overexpresses usually the Epiderml Growth Factor Receptor EGFR [11]. There are no treatment recommendations because of the rarity of this distinct type of breast cancer. Most patients undergo a mastectomy with lymph nodes dissection if possible. Conservation surgery is not usually feasible because of the locally advanced of the disease [12]. Adjuvant Chemotherapy is not usually been used. Rostock et al. suggests that PSCC of the breast is not sensitive to chemotherapeutic agents commonly used for ductal carcinoma such as methotrexate, cyclophosphamide, 5-fluoro-Uracil and anthracycline [13]. Hennessy et al [12] also reported no benefit to neoadjuvant therapy using antracycline/taxane-based regimens, In contrast, a remarkable response was reported in a patient who received neoadjuvant therapy using cisplatin and 5-flouro-Uracil. Our patient had progression after chemotherapy regimen based on 5-fluoro-Uracil and platinium. A good response on metastatic disease has been reported in one patient who received cisplatin and 5-FU, but this has never been investigated in other report [14]. Radiation therapy has been shown little benefit, despite that squamous cell carcinoma are generally radio sensitive [3]. The PSCC of the breast is usually a hormone receptor and HER2 -negative tumor [10] this means that hormone based therapy and HER-2 targeted therapy may not be effective in this tumor. The high frequency of EGFR positivity is interesting [11] the use of anti-EGFR therapy should be explored. The prognosis of this malignancy remains pejorative with a five years overall survival of 51% +/-13% [15] the short period from diagnosis and the death of our patient (7 months) demonstrates that this tumor is very aggressive.

## Conclusion

Primary squamous cell carcinoma of the breast is a rare and aggressive disease often treatment-refractory. Clinical trials including large series of this tumor are needed to increase our knowledge and to improve patient's outcome.
